# A Nine‐Year Diagnostic Odyssey of Refractory Psychiatric Symptoms Resolving After Resection of a CA19‐9‐Producing Ovarian Teratoma: A Case Report and Literature Review

**DOI:** 10.1002/brb3.71370

**Published:** 2026-03-31

**Authors:** Wanli Chen, Xiaoyin Zhuang, Nini Liu, Na Niu, Fei Qiu, Handi Zhang

**Affiliations:** ^1^ Liyuan Hospital, Tongji Medical College Huazhong University of Science and Technology Wuhan China; ^2^ Shantou University Medical College‐Faculty of Medicine of University of Manitoba Joint Laboratory of Biological Psychiatry Shantou University Mental Health Center Shantou China; ^3^ Shanghai Hongkou Mental Health Center Shanghai China

**Keywords:** autoimmune psychosis, biomarker, CA 19‐9, ovarian teratoma, psychiatric symptom

## Abstract

**Background:**

Ovarian teratomas are increasingly recognized as triggers for autoimmune processes, including neuropsychiatric syndromes. This report describes a rare case of isolated autoimmune psychosis associated with an ovarian teratoma and reviews the relevant literature.

**Case Presentation:**

A 15‐year‐old female with a 9‐year psychiatric history was initially diagnosed with schizophrenia. Her symptoms, refractory to multiple antipsychotic regimens, included emotional instability, impulsive aggression, and perceptual abnormalities. Abdominal ultrasonography revealed a giant ovarian teratoma with significantly elevated CA19‐9. Histopathology confirmed neural tissue. Following tumor resection, her psychiatric symptoms resolved dramatically, enabling a return to normal schooling.

**Literature Review:**

Our systematic review identified 32 cases of teratoma‐associated psychosis. Notably, only 2 cases (7.7%) presented without encephalitic features, with one being seronegative for NMDA antibodies. This highlights the exceptional nature of our “pure” psychiatric presentation.

**Conclusions:**

Ovarian teratomas may be associated with suspected cases of isolated autoimmune psychosis, even in the absence of typical neurological signs or positive autoantibodies. In such instances, CA19‐9 elevation could serve as a nonspecific clue. Comprehensive imaging remains crucial for patients with atypical, treatment‐resistant psychosis to avoid diagnostic delays.

## Graphical abstract

A 15‐year‐old with 9 years of treatment‐resistant psychosis achieved rapid remission following resection of a CA19‐9‐producing ovarian teratoma containing neural tissue, highlighting the importance of considering isolated autoimmune psychosis even in seronegative cases and the potential utility of CA19‐9 as a screening biomarker.

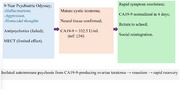

**Abbreviations**
BBB: Blood brain barrier
MCTs: Mature cystic teratomas
MECT: Modified electroconvulsive therapy
NMDA: Anti‐N‐methyl‐D‐aspartate
OT: Ovarian teratoma

## 1 Background

Mature cystic teratomas (MCTs) are ovarian germ cell tumors believed to arise from primordial germ cells due to a failure of meiosis II or from a pro‐meiotic cell in which meiosis has failed (Park et al. 2015). These cells, responsible for transmitting genetic information to offspring during embryonic development (Surti et al. 1990), can give rise to a diverse array of tissues. MCTs are among the most common benign ovarian neoplasms, accounting for 10%–20% of all ovarian tumors (Ayhan et al. 2000). Histologically, they constitute an admixture of well‐differentiated tissues derived from one or more of the three primary germ layers: ectoderm, mesoderm, and endoderm. Consequently, the secretion of various substances from these tissues often leads to the elevation of specific tumor markers (Kyung et al. 2009).

The ectodermal components may include epithelium and neural tissue; mesodermal derivatives can comprise muscle, fat, bone, and cartilage; and endodermal tissues may give rise to structures such as thyroid tissue and gastrointestinal epithelium (Outwater et al. 2001). MCTs are typically slow‐growing and may remain asymptomatic within a patient for many years (Łuczak et al. 2020), often being discovered incidentally during imaging studies or due to hormonal effects after puberty (Cong et al. 2023).

Beyond their well‐described mechanical complications, the potential of MCTs to trigger autoimmune phenomena is of significant clinical relevance. Emerging evidence suggests that specific neural antigens present within ovarian teratomas can elicit a pathogenic immune response (Hong et al. 2021; Vitaliani et al. 2005). Accordingly, psychiatric abnormalities in patients with teratomas have been frequently associated with autoimmune encephalitis (on 2020) and elevated paraneoplastic antibodies (Vitaliani et al. 2005; on 2020).

Herein, we present a compelling case of an adolescent female with a 9‐year diagnostic odyssey across multiple hospitals and specialties. Her case was characterized by atypical psychiatric symptoms that proved refractory to various antipsychotic medications and physical therapies, including electroconvulsive therapy. The pivotal diagnostic clue emerged from an outpatient screening that revealed a giant ovarian teratoma associated with markedly elevated serum CA 19‐9 levels. A dramatic resolution of her psychiatric symptoms followed the surgical resection of the tumor. This case aims to illustrate that the underlying mechanism of her psychiatric presentation is likely related to autoimmune psychosis. We provide a detailed account of the diagnostic and therapeutic journey to foster clinical awareness and further discussion on this rare but important etiology of psychiatric illness. Additionally, we conducted a comprehensive literature review to summarize the clinical features, treatment responses, and prognostic outcomes of psychosis associated with ovarian teratoma. This analysis aims to provide insights to guide future diagnosis and management of this rare and challenging disease.

## 2 Case Presentation

**FIGURE 1:**
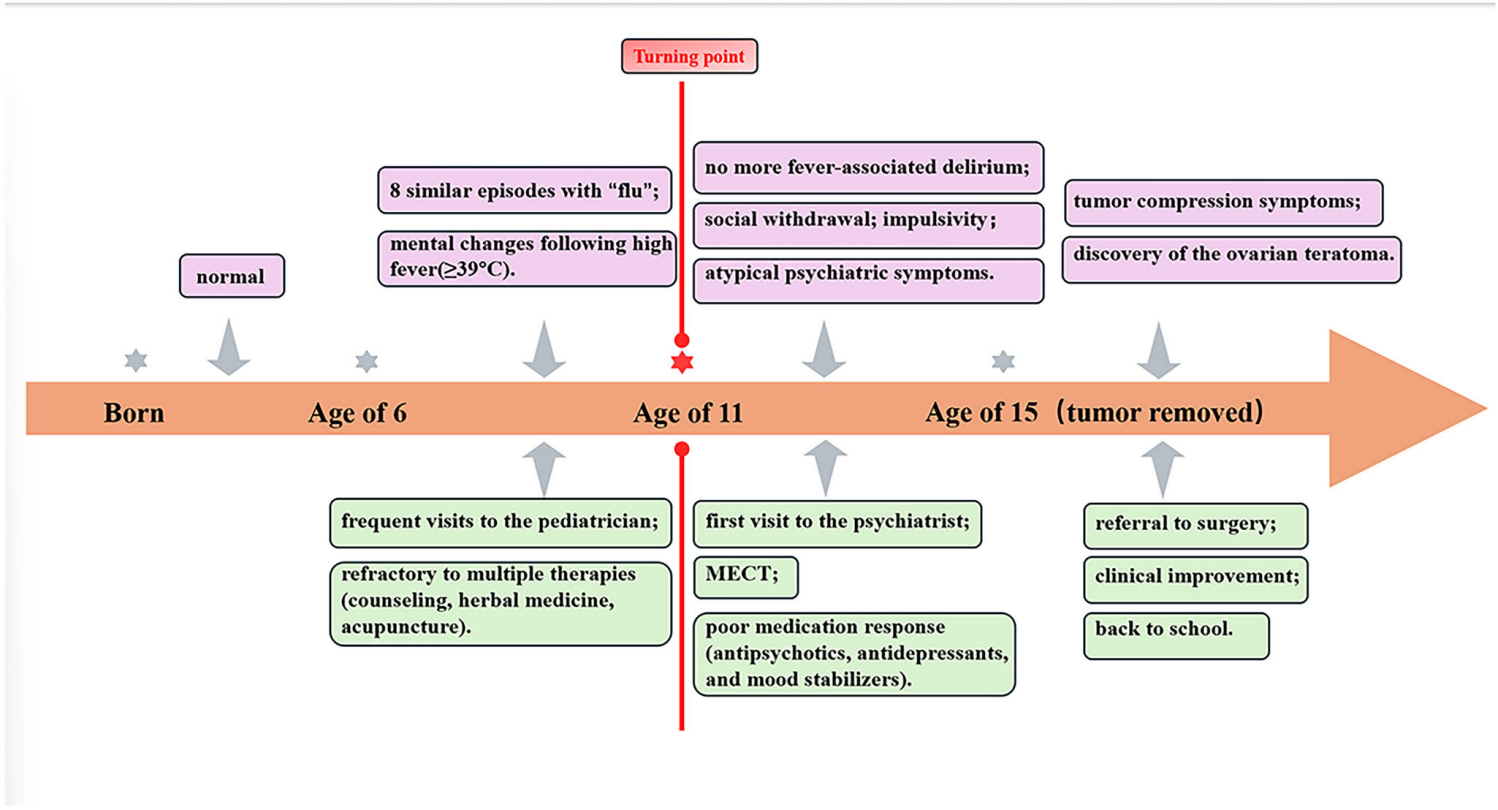
Timeline of the Clinical Course.

Our patient is a 15‐year‐old adolescent female. Her medical history began at the age of 6 when she was first hospitalized in the pediatrics department of a general hospital for “mental changes following high fever” (Figure 1). Her manifestations during these episodes included high fever (≥39°C) accompanied by sleep disturbances, such as suddenly sitting up in bed during the night with an exaggerated and excited expression, waving her hands and feet, and shouting loudly. There was no foaming at the mouth or convulsions of the limbs. Each episode lasted about 10 min and resolved spontaneously, after which the patient had no recollection of the event.

Later, between the ages of 6 and 11, the patient experienced a total of eight similar episodes, each associated with high fever. During these episodes, her speech and behavior were abnormal, presenting with trembling, abnormal excitement or fear, screaming, running around the room, and stating that “someone is chasing me.” The duration varied from 3 to 10 min. During this period, multiple hospitalizations at different institutions included several cranial MRIs, thyroid function tests, and long‐term video electroencephalography (EEG), all of which yielded normal results. Diagnoses were largely “respiratory tract infection” or “tonsillitis,” and treatment involved broad‐spectrum antibiotics followed by discharge upon resolution of fever.

The age of 11 marked a turning point. After the last episode of fever‐associated psychiatric abnormality in July 2021, these acute episodes ceased and have not recurred since. Furthermore, while collecting the medical history, we noted that after the age of 6 (following the onset of fever with psychiatric abnormalities), her behavior and speech were significantly different from her peers. She exhibited intense fear of large red objects, such as lanterns on trees or national flags, requiring her to immediately hide beneath a family member and refusing to look up again. She also demonstrated extreme fear in response to assassination scenes on television, often screaming loudly and fleeing to hide, with a fearful gaze and whole‐body trembling.

After the age of 11, the aforementioned symptoms gradually disappeared. However, she gradually developed sleep disturbances, difficulties maintaining relationships, an inability to understand friends' playful banter and laughter, and a lack of close friends. She became irritable and impulsive in behavior and speech, frequently dismantling her sister's crib, cutting up slippers, and throwing pillows. She displayed intense hostility towards her family members, at times violently assaulting her younger sister. She expressed that “everything around is disgusting and hypocritical,” felt that her parents did not understand her, and, during periods of irritability, stated she “wanted to kill them immediately” and “hoped her parents would die right away.” Her family took her to various hospitals for treatments, including psychological counseling, traditional Chinese herbal medicine, and acupuncture, all of which showed no improvement.

In October 2023, the patient's condition deteriorated acutely, culminating in a knife threat against family members that necessitated emergency psychiatric hospitalization. Upon admission, a diagnosis of schizophrenia was considered. Comprehensive physical and auxiliary examinations, including cranial MRI, routine blood and urine analyses, thyroid function, and sex hormone profiles, were within normal limits. Initial pharmacotherapy with magnesium valproate (500 mg/day) and aripiprazole (2.5 mg/day) was ineffective.

Due to persistent severe agitation, screaming, physical aggression (e.g., door‐banging), food refusal, and suicidal ideation, she underwent a course of eight sessions of modified electroconvulsive therapy (MECT). For the first six sessions, the stimulus dose was set at 10%, which was subsequently increased to 15% for the final two sessions. Anesthesia was induced with etomidate, and muscle relaxation was achieved with succinylcholine. Throughout the treatment course, static impedance ranged from 1130 to 2320 Ω, dynamic impedance was maintained at 200 Ω, and electrode contact remained consistently adequate. The seizure adequacy rate reached 100% across all treatments, with EEG‐monitored seizure duration ranging from 80 to 160 s. Both the average seizure energy index (ASEI) and the postictal suppression index (PSI) demonstrated adequate seizure quality, confirming that all treatment sessions met the criteria for therapeutic efficacy. This intervention resulted in marginal improvement, sufficient only to establish limited cooperation. At this point, she reported auditory hallucinations consisting of derogatory voices, persistent feelings of being monitored, and a belief that going outside was unsafe. She further described intrusive, distressing mental imagery of bestiality and orgies, which she found repulsive and which led her to believe she was “a pervert.”

Pharmacological management was subsequently intensified and modified. A regimen of magnesium valproate sustained‐release tablets (1000 mg/day), risperidone oral solution (1.5 mL/day), and bupropion sustained‐release tablets (300 mg/day) was initiated but yielded minimal clinical benefit. The patient continued to exhibit violent rage, homicidal thoughts, poor concentration, compulsive spending, and persistent suicidal impulses. A further switch to a combination of magnesium valproate (1000 mg/day), aripiprazole (5 mg/day), and escitalopram (10 mg/day) led to a modest reduction in auditory hallucinations; however, her core impulsive and aggressive behaviors remained profoundly unchanged.

A pivotal diagnostic breakthrough occurred in June 2025. The evaluation of new‐onset urinary incontinence led to an abdominal ultrasonography, which identified a large (6 × 7 × 6 cm) left ovarian teratoma. Following gynecological consultation, surgical resection was recommended. Preoperative laboratory tests revealed a significantly elevated serum CA19‐9 level of 332.5 U/ml (reference range: ≤34 U/ml). Other tumor markers—including AFP, CEA, CA‐125, SCC, CA15‐3, CA72‐4, and HCG—were all within normal ranges. Thyroid function tests and thyroid‐related antibodies (TgAb, TPOAb, and TRAb) were also unremarkable. Notably, in the 48 h preceding the surgery, the patient's behavioral dysregulation escalated to a peak, characterized by severe emotional lability, compulsive spending, and agitation, culminating in her absconding from home on the eve of the operation, necessitating police intervention for her safe return.

Remarkably, a pronounced stabilization in her affect and behavior was observed immediately following tumor resection. Histopathological examination confirmed the lesion to be a mature, benign cystic teratoma. The serum CA 19‐9 level normalized to within the reference range within four days postoperatively, paralleling her clinical improvement.

Given the strong temporal association between tumor resection and symptomatic improvement, a paraneoplastic or autoimmune etiology was strongly suspected. Subsequent investigations, including serum anti‐NMDA receptor antibodies, C‐reactive protein (CRP), and contrast‐enhanced cranial MRI, returned normal results. Lumbar puncture for cerebrospinal fluid analysis was declined by the family.

**FIGURE 2:**
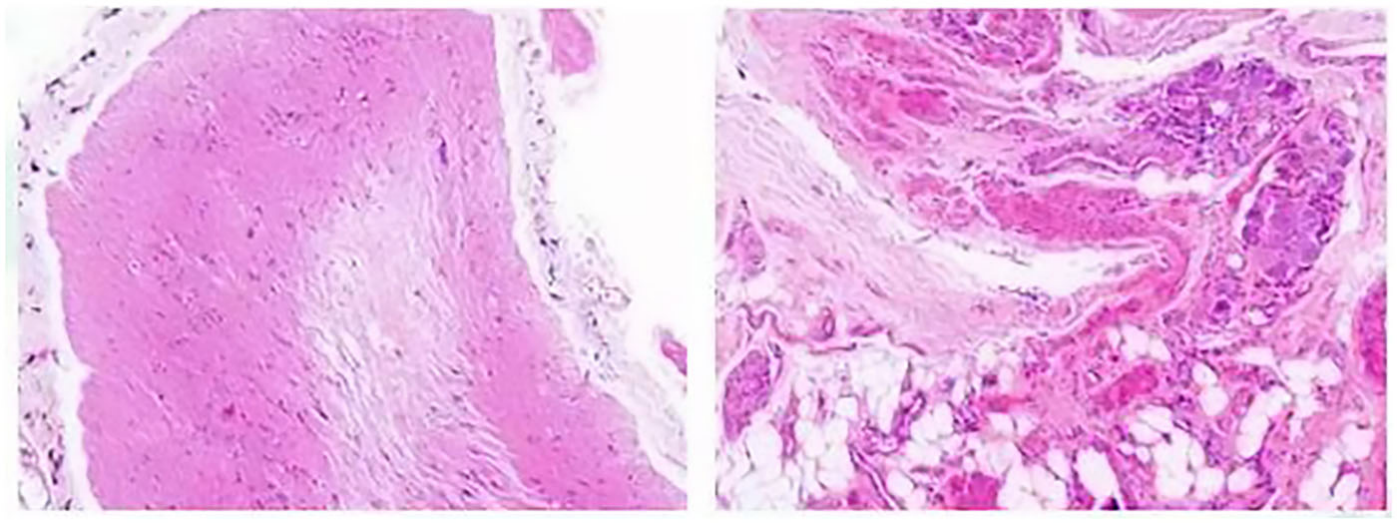
Histopathology of the ovarian mature cystic teratoma showing neural tissue components.

The patient's postoperative clinical course was marked by sustained and significant improvement. By the 8‐week follow‐up, she no longer exhibited overt psychotic symptoms, aggressive behaviors, or suicidal ideation. She demonstrated a renewed capacity for socialization and successfully reintegrated into school. Standardized psychiatric assessments showed progressive score reductions, consistent with her observed recovery. Further histopathological analysis, initiated following clinical correlation, confirmed the presence of mature neural elements within the teratoma, including brain tissue, glial cells, and ganglion cells (Figure 2).

**FIGURE 3:**
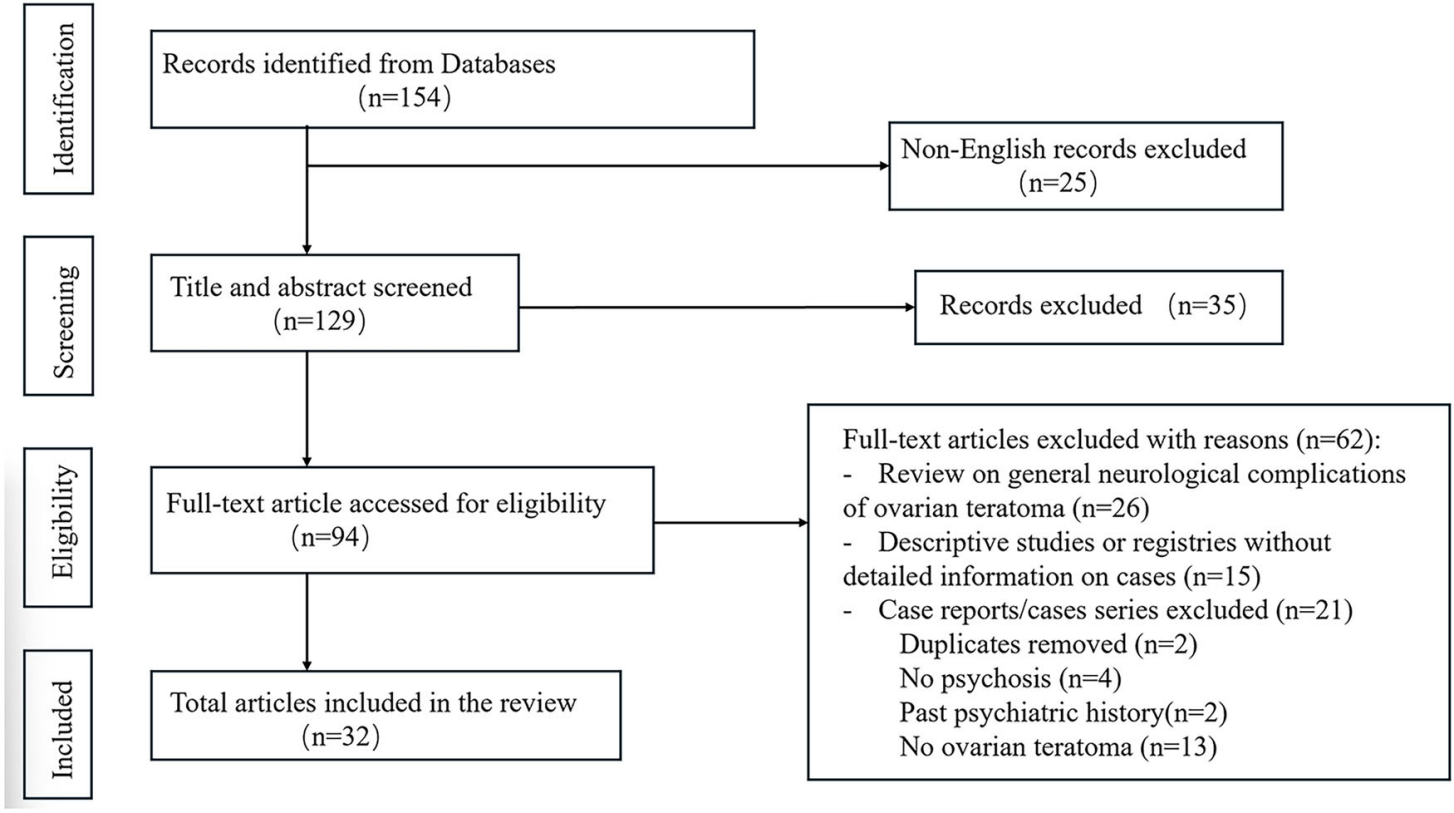
Flowchart showing our literature search.

During the 3‐month postoperative follow‐up, she displayed no neurological deficits and had resumed regular school attendance. Her psychotropic medication regimen was successfully tapered to a maintenance dose of aripiprazole (5 mg/day) and magnesium valproate (500 mg/day), with a planned gradual reduction based on continued stability (Figure 3). According to the most recent outpatient follow‐up record (March 8, 2026), the patient's pharmacological regimen has been reduced to aripiprazole monotherapy at a dose of 2.5 mg daily. The patient self‐reported a high degree of emotional stability; however, she acknowledged that, owing to her prolonged history of illness, she has been estranged from normative social environments for an extended period. Consequently, she occasionally experiences mild sensitivity and suspiciousness in interpersonal interactions. Her parents expressed considerable satisfaction with her recovery, noting a marked improvement in her initiative and proactive engagement. Notably, she recently succeeded in selling several of her comic works online, thereby generating a modest personal income. As a precautionary measure, the outpatient physician has opted to maintain a low dose of aripiprazole. Nevertheless, given her current trajectory, there is optimism regarding the possibility of complete drug discontinuation within the forthcoming year. Informed consent for publication of this case report was obtained from the patient and her parents.

### 2.1 Literature Review

#### 2.1.1 Methods

**FIGURE 4:**
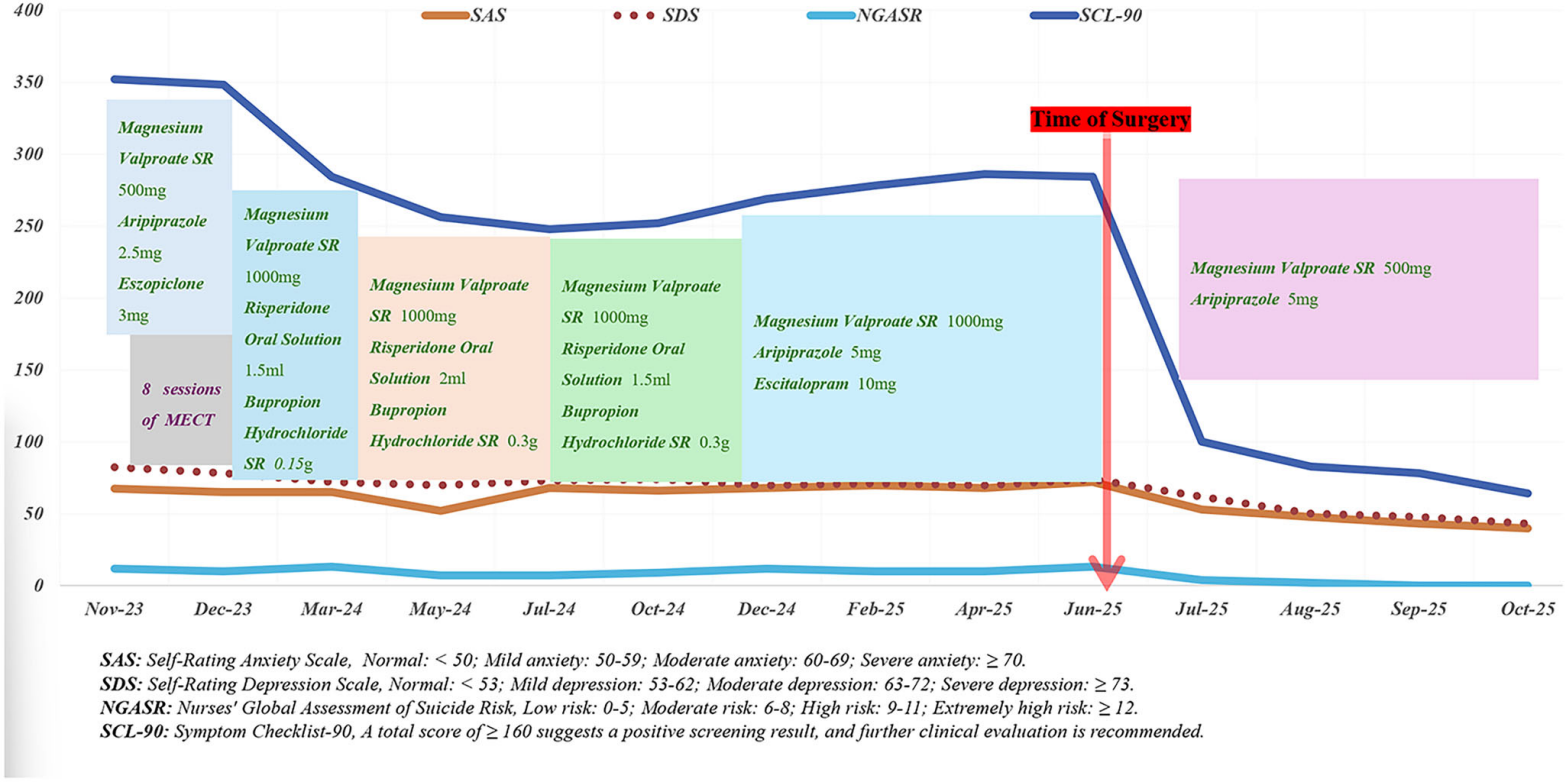
Trends in Patient Scale Scores.

We performed a systematic review of the literature to identify all well‐documented cases of ovarian teratoma‐associated psychosis. A search was conducted on November 1, 2025, by using PubMed/MEDLINE, Google Scholar, Scopus, EBSCO, and DOAJ; the following search terms were used: “Ovarian Teratoma” OR “Teratoma of Ovary” OR “Dermoid cyst of the ovary” AND “Psychosis,” limited to publications in English. The study flow is described in Figure 4. We included studies that met the following inclusion criteria: (1) Patients diagnosed with ovarian teratoma and incident psychosis; (2) Articles identified as case reports, case series, letters to the editor, correspondences, and commentaries describing patient presentations. The methodological quality of each case report was evaluated according to the Joanna Briggs Institute (JBI) critical appraisal checklist (Munn et al. 2020). The assessment concluded that all reports complied with reporting standards, and there was no evidence that conflicts of interest impacted the validity of our findings.

### 2.2 Literature Review

#### 2.2.1 Results

**FIGURE 5:**
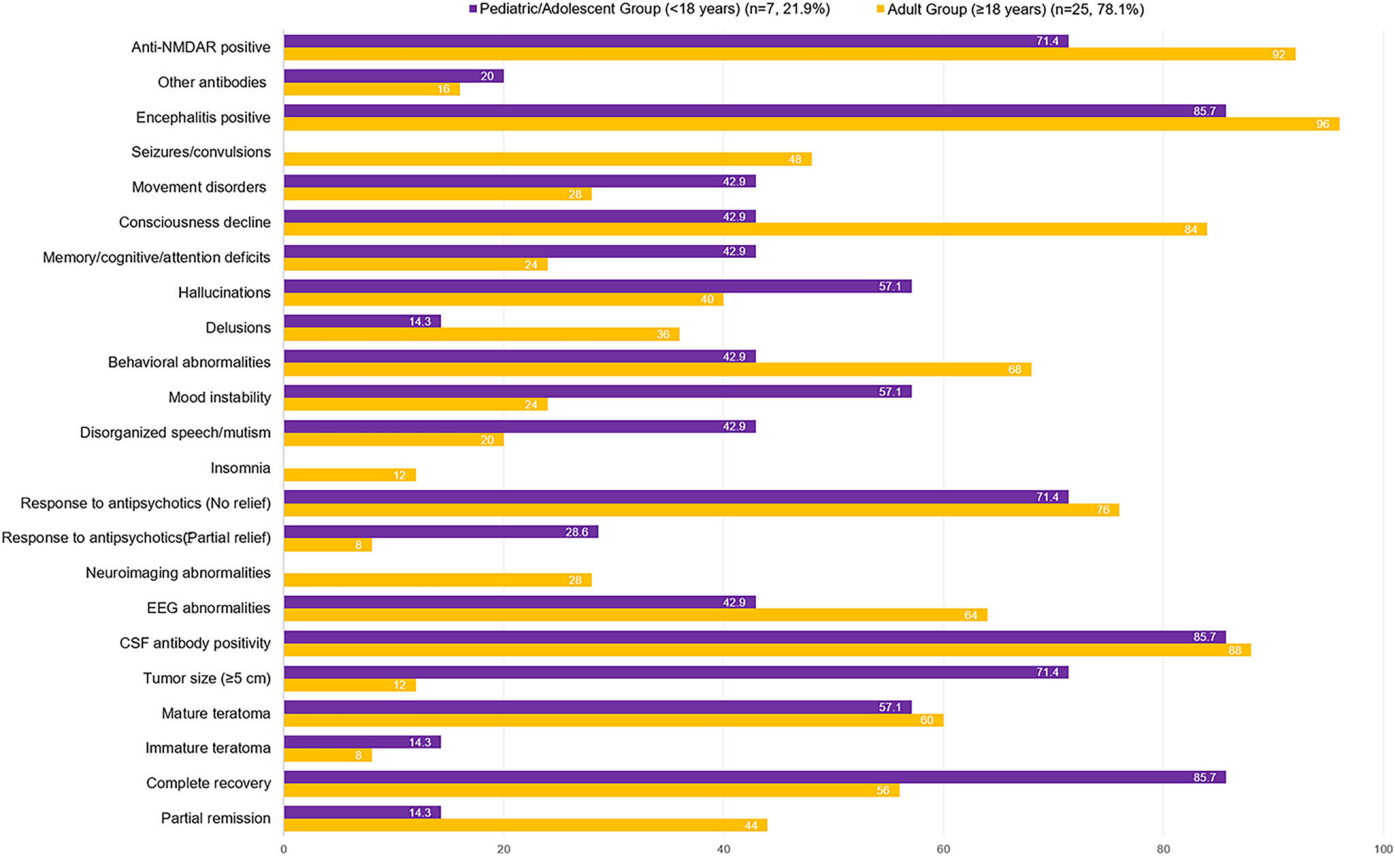
Age‐stratified clinical characteristics in ovarian teratoma patients with neuropsychiatric symptoms.

As shown in Figure 4, a total of 29 articles (Herrmann et al. 2020) reporting on 32 patients were included in the analysis (Table 1). We used age as the core grouping variable to compare the clinical characteristics of patients in different age groups, comprising 7 (21.9%) patients in the pediatric/adolescent group (<18 years) and 25 (78.1%) patients in the adult group (≥18 years) (Figure 5). Regarding antibody types, the majority of patients demonstrated anti‐NMDAR positivity. Specifically, anti‐NMDAR antibodies were detected in 5 (71.4%) patients in the pediatric/adolescent group and 23 (92.0%) patients in the adult group. Other antibodies (including CASPR2, AFP, and AMPAR) were observed in 1 (20.0%) patient in the younger group and 4 (16.0%) patients in the adult group. Clinical encephalitis was present in the vast majority of cases, reported in 6 (85.7%) pediatric/adolescent patients and 24 (96.0%) adult patients.

**TABLE 1:** Summary of clinical features in ovarian teratoma‐associated psychosis cases.

Ref	Age		Antibody type	Encephalitis		Neurological deficits	Psychiatric symptoms	Response to antipsychotics	Neuroimaging	EEG	CSF	Disease duration (months)	Tumor size (cm)	Histopathology	Outcome
Herrmann et al. (2020)	53	CASPR2		—	Deficits in concentration, attention and short‐term memory		Depression, suicidal thoughts, delusions of persecution	Not relieved	—	—	—	6	2.5 × 2.5	NA	Complete recovery
Nokura et al. (1997)	19		AFP	+	Roving eye movements, central hypoventilation		Confusional state of schizophrenia.	Not relieved	+	+	—	2	8 × 8	Immature	Partial remission
Lee et al. (2003)	15		CA125	+	Rest tremors and generalized rigidity.		Auditory hallucinations, talking nonsense	Not relieved	—	+	—	1	20 × 13 × 8 12 × 6 × 7	Mature	Partial remission
Sun et al. (2023)	37		Anti‐NMDA‐R Anti‐AMPAR2	+	Movement disorder, dystonia, and epilepsy		Slow reaction, behavior change and disorientation mutism	Not relieved	—	+	Anti‐NMDA‐R antibodies Anti‐AMPAR2 antibodies	3	1.1 × 2.2	Mature	Complete recovery
Fawcett (2010)	28		Anti‐NMDA‐R CA125	+	Seizure and staring into space		Hyperactivity; hypergraphia, loquaciousness	Not relieved	—	+	Anti‐NMDA‐R antibodies	2	NA	NA	Partial remission
Curnow et al. (2019)	14		Anti‐NMDA‐R	—	—		Thought disorder, paranoia, and auditory hallucinations	Partial relieved	—	+	Anti‐NMDA‐R antibodies	1	NA	NA	Complete recovery
Voice et al. (2017)	17		Anti‐NMDA‐R	+	Choreoathetoid movements and lingual facial dyskinesia		Extremely agitated, emotional outbursts, periods of aggression	Not relieved	NA	NA	Anti‐NMDA‐R antibodies	1	4 × 6	Mature	Complete recovery
Liu et al. (2015)	22		Anti‐NMDA‐R	+	Headache, confusion, seizures and a stiff neck		Behavior change, aggression	NA	—	+	Anti‐NMDA‐R antibodies	1.5	1.4 × 1.3	Mature	Complete recovery
Liu et al. (2015)	31		Anti‐NMDA‐R	+	Seizures and agitation		Confusion, agitation, and auditory hallucinations	NA	—	+	Anti‐NMDA‐R antibodies	1	2.3 × 1.5	Mature	Complete recovery
Chiu et al. (2019)	27		Anti‐NMDA‐R	+	Consciousness change and seizure		Abnormally aggressive behavior	NA	—	—	Anti‐NMDA‐R antibodies	1	2.0 × 1.0	NA	Partial remission
Chiu et al. (2019)	28		Anti‐NMDA‐R	+	Confusion, dull response and acute disorientation		Consciousness disturbance with making faces	Not relieved	+	+	—	1	3.3 × 3.0	Mature	Complete recovery
Aung et al. (2017)	16		Anti‐NMDA‐R	+	Echolalia, orofacial dyskinesias, choreoathetosis, catatonia		Aggression, auditory hallucinations, suicidal thoughts	Not relieved	—	—	Anti‐NMDA‐R antibodies	1	6.6 × 4.9	NA	Complete recovery
Liang et al. (2017)	17		Anti‐NMDA‐R	+	Unresponsive, impaired ability of calculating and memorizing		Excitement, speech confusion	Not relieved	—	—	Anti‐NMDA‐R antibodies	1	1.4 × 1.2	Mature	Complete recovery
Liang et al. (2017)	16		Anti‐NMDA‐R	+	Unable to urinate or defecate, limb twitching and slow reaction		Abnormal behavior and confused speech	Partial relieved	—	+	Anti‐NMDA‐R antibodies	2	12.7 × 12.5	Immature	Complete recovery
Câmara‐Pestana et al. (2022)	27		Anti‐NMDA‐R	+	Psychomotor slowing, defects in delayed evocation, attention deficit		Suspicion, disorganized thought, delusions, auditory hallucinations, insomnia	Not relieved	+	+	Anti‐NMDA‐R antibodies	1	NA	NA	Partial remission
Imai et al. (2015)	39		Anti‐NMDA‐R	+	Deteriorated emotional lability, increased disorientation, progressive dyskinesia		Hallucinations and emotional lability	Not relieved	—	—	Anti‐NMDA‐R antibodies	2	3.0 × 2.3	Mature	Complete recovery
Mateus et al. (2025)	30		Anti‐NMDA‐R	+	Fluctuation in consciousness, lack of response to verbal stimuli		Significant emotional lability, disorganized speech, and restlessness, auditory hallucinations	Partial relieved	+	—	Anti‐NMDA‐R antibodies	2	1.8 × 1.8	Mature	Complete recovery
Jarmoc et al. (2024)	32		Anti‐NMDA‐R	+	Definitive seizure		Delusional thought content and agitation, catatonia	Partial relieved	—	+	Anti‐NMDA‐R antibodies	2	3.7 × 2.9	Mature	Partial remission
Wali et al. (2011)	29		Anti‐NMDA‐R	+	Memory deficit, fluctuations in alertness, orientation and concentration		Auditory and visual hallucinations agitated and violent	Not relieved	—	+	Anti‐NMDA‐R antibodies	1	2 × 5 × 5	Immature	Partial remission
Shimoyama et al. (2016)	19		Anti‐NMDA‐R	+	Repeated generalized seizures, memory deficit		Acute psychosis, emotionally lability, behavioral changes	Not relieved	—	+	Anti‐NMDA‐R antibodies	2.5	NA	NA	Complete recovery
Lwanga et al. (2018)	26		Anti‐NMDA‐R	+	Seizures, stereotypic postures		Staring spells, auditory hallucinations, and insomnia	Not relieved	+	+	Anti‐NMDA‐R antibodies	2	0.8 × 0.8	Mature	Complete recovery
Lwanga et al. (2018)	23		Anti‐NMDA‐R	+	Seizures, fast unintelligible speech, inability to follow commands		Hallucinations and bizarre thoughts	Not relieved	—	+	Anti‐NMDA‐R antibodies	3	0.6 × 0.6	Mature	Partial remission
Koksal et al. (2015)	25		Anti‐NMDA‐R	+	Confusion, cog‐wheel rigidity, generalized seizure		Insomnia, agitation, irritability and delusions and hallucinations	Not relieved	—	—	Anti‐NMDA‐R antibodies	1	NA	NA	Partial remission
Hegen et al. (2016)	30		Anti‐NMDA‐R	+	Short‐term amnesia, complex epileptic seizures		Bizarre behaviors, delusion, agitation and mutism	Not reported	+	—	Anti‐NMDA‐R antibodies	3	NA	NA	Partial remission
Omura et al. (2015)	20		Anti‐NMDA‐R	+	Neck stiffness, tremulous arms, facial dyskinesia		Hallucinations, agitation, and confusion	Not relieved	—	+	Anti‐NMDA‐R antibodies	1	NA	NA	Complete recovery
Pavǎl et al. (2022)	24		Anti‐NMDA‐R	+	Short‐term memory deficits		Delusions of pregnancy, visceral hallucinations and bizarre behavior	Not relieved	—	—	Anti‐NMDA‐R antibodies	2	NA	Mature	Complete recovery
Casarcia et al. (2024)	39		Anti‐NMDA‐R	+	Fatigued, slow speech rate with an irregular rhythm		Confusion, disorientation, hallucinations, paranoia	Not relieved	—	+	Anti‐NMDA‐R antibodies	1	1.6	Mature	Complete recovery
Molina et al. (2025)	34		Anti‐NMDA‐R	+	Overt excitement		Agitation and mania	Not relieved	—	—	Anti‐NMDA‐R antibodies	1	1.3 × 0.6	Mature	Complete recovery
Gharedaghi et al. (2018)	26		Anti‐NMDA‐R	+	Dystonic movements, rigidity of the upper and lower extremities, axial spasticity		Bizarre behavior, paranoia and delusion	Not relieved	+	—	Anti‐NMDA‐R antibodies	1	11	Mature	Partial remission
Braverman et al. (2015)	12		Anti‐NMDA‐R	+	Constricted and minimally reactive pupils, Unable to respond to commands, unable to swallow, and incontinent		Auditory hallucinations, emotionally Labile and confused	Not relieved	—	NA	Anti‐NMDA‐R antibodies	2	11 × 6 × 8	Mature	Complete recovery
Hayashi et al. (2014)	18		Anti‐NMDA‐R	+	Involuntary movements, disturbance of consciousness, and central hypoventilation		Violent behavior and speech disabilities	Not relieved	NA	+	Anti‐NMDA‐R antibodies	1	0.5 × 0.7	Mature	Partial remission
Andaya and Diaz (2024)	25		Anti‐NMDA‐R	+	Epilepticus, orofacial dyskinesias		Catatonia, delusions, restlessness, agitations, and thoughts of persecution	Not relieved	—	+	Anti‐NMDA‐R antibodies	3	2.6 × 3.2 × 3.9	Mature	Complete recovery

*Note*: Disease duration was defined as the time from symptom onset to hospital discharge accompanied by symptom resolution. Abbreviations: Anti‐NMDA‐R, anti‐N‐methyl‐D‐aspartate receptor;‐, negative; +, positive; NA, not applicable.

With respect to neurological deficits, notable differences were observed between the groups. Consciousness decline/lethargy/reduced responsiveness was the most prevalent neurological finding, occurring in 21 (84.0%) adult patients, compared to 3 (42.9%) in the pediatric/adolescent group. Seizures/convulsions were reported exclusively in the adult group, affecting 12 (48.0%) patients, while no pediatric/adolescent patients presented with this symptom (0.0%). Movement disorders (chorea/rigidity/involuntary movements) were observed in 3 (42.9%) younger patients and 7 (28.0%) adult patients. Memory/cognitive/attention deficits were present in 3 (42.9%) patients in the pediatric/adolescent group and 6 (24.0%) in the adult group.

Regarding psychiatric manifestations, the frequency and distribution varied between age groups. In the pediatric/adolescent group, the most frequently reported symptoms were hallucinations and mood instability/depression/anxiety, each occurring in 4 (57.1%) patients. In contrast, among adult patients, behavioral abnormalities/agitation/aggression was the predominant psychiatric symptom, reported in 17 (68.0%) patients. Hallucinations were reported in 10 (40.0%) adult patients. Delusions/paranoia/suspiciousness were observed in 1 (14.3%) pediatric/adolescent patient and 9 (36.0%) adult patients. Disorganized speech/mutism was present in 3 (42.9%) younger patients and 5 (20.0%) adult patients. Sleep disturbances were reported exclusively in the adult group, affecting 3 (12.0%) patients.

Concerning treatment response, the majority of patients in both groups showed no relief with antipsychotic medication. No relief was reported in 5 (71.4%) pediatric/adolescent patients and 19 (76.0%) adult patients. Partial relief was observed in 2 (28.6%) younger patients and 2 (8.0%) adult patients. Response data were not reported for 4 (16.0%) adult patients. Regarding ancillary investigations, CSF antibody positivity was the most common abnormal finding, present in 6 (85.7%) pediatric/adolescent patients and 22 (88.0%) adult patients. EEG abnormalities were observed in 3 (42.9%) younger patients and 12 (64.0%) adult patients. Neuroimaging abnormalities were reported exclusively in the adult group, affecting 7 (28.0%) patients.

A striking difference was observed in tumor size between the two groups. In the pediatric/adolescent group, the majority of patients (5, 71.4%) presented with large tumors (≥5 cm), whereas only 3 (12.0%) adult patients had tumors of this size. Tumor size was not reported for 1 (14.3%) pediatric/adolescent patient and 7 (28.0%) adult patients. Regarding histopathological type, mature teratoma was the most common finding in both groups, present in 4 (57.1%) pediatric/adolescent patients and 15 (60.0%) adult patients. Immature teratoma was observed in 1 (14.3%) younger patient and 2 (8.0%) adult patients. Pathological data were not reported for 2 (28.6%) patients in the pediatric/adolescent group and 8 (32.0%) in the adult group. With respect to prognosis, complete recovery was achieved in the majority of pediatric/adolescent patients (6, 85.7%), compared to 14 (56.0%) adult patients. Partial remission was observed in 1 (14.3%) younger patient and 11 (44.0%) adult patients.

## 3 Discussion

### 3.1 Consistency With Previous Studies

This case demonstrates significant alignment with previous literature across several key dimensions: the patient belongs to the core affected demographic of young females and exhibited the characteristic treatment‐resistant nature of the disorder; psychiatric symptoms, including hallucinations, delusions, and psychomotor excitement, were entirely consistent with the core phenotypic spectrum of the condition. Despite negative serum antibodies, the presence of neural tissue within the tumor provides a common pathological basis for autoimmune pathogenesis. Most importantly, the dramatic symptomatic resolution following tumor resection underscores the fundamental importance of etiological treatment, while repeatedly normal neuroimaging findings further highlight the recognized diagnostic limitations of imaging in this disease category. These consistencies collectively affirm that our case represents a valid component of the disease spectrum, with its distinctiveness firmly rooted in these established commonalities.

### 3.2 Unique Features of This Case

This case underscores the necessity of including paraneoplastic and immune‐mediated mechanisms in the differential diagnosis of first‐episode psychosis, particularly in cases exhibiting red‐flag symptoms such as refractoriness to standard antipsychotic pharmacotherapy. To our knowledge, this is the first reported case of an adolescent female with isolated psychiatric symptoms secondary to a giant ovarian teratoma associated with a significantly elevated CA19‐9 level. The patient's 9‐year diagnostic course, culminating in the discovery and resection of the tumor followed by dramatic symptom resolution, offers profound insights, even in the absence of definitive autoimmune serological markers. The rapid normalization of serum CA19‐9 within 4 days post‐operatively, paralleling the striking clinical improvement, provides compelling evidence for a pathophysiological link between the teratoma and the psychiatric syndrome. The possible pathophysiological mechanisms underlying her psychiatric presentation are explored as follows.

#### 3.2.1 Autoimmune Mechanisms not Covered by Routine Assays

This mechanism represents the pathophysiological explanation most consistent with the existing literature. Although serum testing for conventional antibodies, including anti‐NMDAR, was negative, histopathological examination of the resected teratoma confirmed the presence of mature neural tissue, including brain tissue, glial cells, and ganglion cells. This finding provides a definitive anatomical substrate for an immune response directed against neural antigens (Surti et al. 1990; Outwater et al. 2001; Cong et al. 2023; Tan and Zhang 2024). Potential explanations for the negative antibody findings include the following: (1) antibodies confined to the cerebrospinal fluid with seronegativity (intrathecal synthesis), a possibility that could not be excluded as lumbar puncture was declined by the family; (2) antibodies targeting rare neuronal antigens not tested; and (3) a pathological process predominantly mediated by T‐cell‐mediated cellular immunity rather than humoral immunity, a phenomenon documented in paraneoplastic syndromes that often presents with seronegativity (Vitaliani et al. 2005). The rapid postoperative symptomatic improvement, coinciding with removal of the persistent immune stimulus (neural tissue within the teratoma), renders this mechanism one of the most plausible explanations.

#### 3.2.2 Integrative Consideration of Multifactorial Mechanisms

In addition to the core autoimmune mechanism discussed above, the teratoma may contribute to the onset and evolution of psychiatric symptoms through several alternative pathways. First, the remote effects of the tumor warrant consideration: its diverse components exhibit an active secretory function (Ayhan et al. 2000; Kyung et al. 2009), as evidenced by the patient's markedly elevated CA19‐9 levels, which normalized rapidly after surgery. It is plausible that secreted products—such as inflammatory cytokines, neuropeptides, or other bioactive substances—enter the central nervous system through areas with a compromised blood‐brain barrier (Langen et al. 2019; Serlin et al. 2015), thereby influencing neurotransmitter metabolism. Given the patient's long‐standing refractory psychiatric symptoms, it would be valuable to explore the therapeutic potential of metabolic interventions—particularly the ketogenic diet—in this context. The ketogenic diet has been shown to exert neuroprotective effects through multiple mechanisms, including inhibition of neuroinflammation, reduction of reactive oxygen species, and enhancement of mitochondrial biogenesis, which may be relevant to the inflammatory and metabolic perturbations associated with teratoma‐related psychiatric syndromes (Uchida et al. 2017; Zhuang et al. 2025). In this context, the patient may have a genetic predisposition to psychiatric illness or a neurodevelopmental vulnerability, a basis that can be inferred from her history of abnormal febrile delirium episodes during childhood (Paus et al. 2008). The immune or metabolic perturbations associated with the teratoma may not directly “cause” the psychosis but rather modify and exacerbate the course of this underlying predisposition; the continued need for low‐dose psychiatric medication after surgery supports this background factor. Additionally, the large teratoma (6 × 7 × 6 cm) could have influenced the local endocrine environment through a pelvic mass effect or paracrine mechanisms (Nguyen et al. 2007; Sasi et al. 2014), particularly during the vulnerable period of adolescence. However, a simple mass effect is unlikely to account for complex psychotic symptoms and is more plausibly considered an aggravating factor rather than the primary etiology. It is important to note that while the manuscript highlights the significant symptom improvement following tumor resection, the literature indicates that not all teratoma‐associated cases achieve complete post‐operative remission (Reddihalli et al. 2015; Tantitamit et al. 2020; Uchida et al. 2018). Indeed, failure to improve after ovarian resection could, in some instances, signal recurrent teratoma or residual disease (Uchida et al. 2018), underscoring the need for careful post‐operative monitoring in refractory cases. Although the placebo effect of a significant surgical intervention cannot be entirely ruled out, the nature of the postoperative improvement—its immediacy, sustainability, a substantial decrease in patient scale scores (Figure 3), and its temporal correlation with the normalization of CA19‐9 levels—represents objective evidence that extends far beyond what a mere psychological effect could explain (Gaab 2019; Pacheco‐López et al. 2006). When interpreting autoantibody results in autoimmune neuropsychiatric syndromes, caution is required if IVIg has been administered, as it leads to passive immunoglobulin transfer and may confound interpretation (Grüter et al. 2020; Uchida et al. 2017). Although our patient did not receive IVIg prior to testing, this consideration remains relevant for similar cases where immunotherapy precedes diagnostic evaluation.

#### 3.2.3 Isolated Autoimmune Psychosis: Expanding the Phenotypic Spectrum

A second critical aspect of this case is the exclusively psychiatric presentation in the absence of classical neurological signs. This contrasts sharply with the typical presentation of anti‐NMDAR encephalitis associated with ovarian teratomas, which features a prominent constellation of neurological symptoms (e.g., seizures, dyskinesia, autonomic instability) (Dalmau et al. 2008; Dalmau et al. 2007; Jang et al. 2024; Titulaer et al. 2013). Our patient's “pure” psychiatric phenotype—persisting for years without neurological deterioration—aligns with the recently proposed diagnostic concept of “autoimmune psychosis” (Pollak et al. 2020). This term distinguishes cases with predominant psychotic symptoms and minimal neurological features from full‐blown autoimmune encephalitis. Although serum anti‐NMDAR antibodies and other standard paraneoplastic panels were negative in our patient, this does not rule out an autoimmune mechanism. Antibodies may have been confined to the cerebrospinal fluid (which was not tested per family refusal), or they may be directed against neuronal targets not included in commercial assays (Moldavski et al. 2021). The presence of mature neural tissue within the resected teratoma provides a plausible anatomical substrate for an ongoing pathogenic immune response (Dalmau et al. 2008; Tüzün et al. 2009).

#### 3.2.4 CA 19‐9 as a Serological Clue to an Underlying Organic Etiology

Third, this case highlights the potential utility of the tumor marker CA 19‐9 as a valuable biomarker in the workup of refractory psychosis. The significant preoperative elevation of CA 19‐9 and its rapid normalization following tumor resection provide compelling indirect evidence of its relevance. In mature cystic teratomas (MCTs), CA 19‐9 is typically produced by gastrointestinal‐type epithelial components within the tumor (Atabekoğlu et al. 2005; Dede et al. 2006; Ustunyurt et al. 2009). Its elevation, particularly in large tumors, is a recognized phenomenon. In this context, an unexplained elevation of CA 19‐9 in a young female with atypical psychosis should raise a high index of suspicion for an underlying MCT. While CA 19‐9 itself is not a direct marker of neural tissue or autoimmune activity, it serves as a sensitive surrogate marker for the presence of a teratoma. Given that such teratomas can harbor neuroglial elements (as confirmed in our case), the marker effectively flags patients at risk for paraneoplastic/autoimmune psychiatric syndromes. This inexpensive and readily available test could thus serve as a crucial screening tool, prompting further imaging and specialist consultation.

## 4 Conclusions

This study, integrating a suspected case of isolated autoimmune psychosis associated with a CA19‐9‐producing ovarian teratoma with a systematic review of 29 published cases, delineates a unique clinical phenotype within the teratoma‐associated neuropsychiatric spectrum. Our case specifically demonstrates the clinical utility of CA19‐9 as an accessible serological marker for identifying underlying teratomas in patients presenting with atypical, seronegative psychiatric syndromes without classic encephalitic features. Collectively, our findings confirm that while most teratoma‐associated neuropsychiatric disorders manifest as encephalitic syndromes, a distinct minority present with isolated psychotic features.

Based on our case experience and literature synthesis, we emphasize the critical importance of systematic somatic evaluation—including pelvic imaging and tumor marker assessment—in the workup of treatment‐resistant psychosis. We strongly advocate for enhanced clinical awareness of organic etiologies and promote multidisciplinary collaboration across psychiatry, neurology, and gynecology to facilitate early recognition and targeted intervention. Future efforts should focus on elucidating the underlying immunopathological mechanisms and establishing standardized management protocols incorporating tumor screening, comprehensive antibody testing, and immunomodulatory strategies to ultimately improve long‐term patient outcomes.

## Author Contributions

**Wanli Chen: **conceptualization, software, validation, investigation, resources, and writing – original draft. **Handi Zhang: **conceptualization, methodology, validation, formal analysis, investigation, writing – review & editing, and supervision. **Xiaoyin Zhuang**: methodology, resources, and data curation. **Nini Liu: **methodology and software. **Na Niu**: validation and visualization. **Fei Qiu**: resources and visualization. All contributors provided feedback on the drafts. The final manuscript was read and approved by all authors.

## Funding

The authors have nothing to report.

## Ethics Statement

Consent was provided by the patient and her parents to enable anonymized reporting of the results of this study.

## Conflicts of Interest

The authors declare no conflicts of interest.

## Data Availability

Data will be made available on request.
